# Ontogeny of Metabolic Rate and Red Blood Cell Size in Eyelid Geckos: Species Follow Different Paths

**DOI:** 10.1371/journal.pone.0064715

**Published:** 2013-05-21

**Authors:** Zuzana Starostová, Marek Konarzewski, Jan Kozłowski, Lukáš Kratochvíl

**Affiliations:** 1 Department of Zoology, Faculty of Science, Charles University in Prague, Prague, Czech Republic; 2 Department of Animal Ecology, University of Białystok, Białystok, Poland; 3 Mammal Research Institute, Polish Academy of Sciences, Białowieża, Poland; 4 Institute of Environmental Sciences, Jagiellonian University, Kraków, Poland; 5 Department of Ecology, Faculty of Science, Charles University in Prague, Prague, Czech Republic; University of Hamburg, Germany

## Abstract

While metabolism is a fundamental feature of all organisms, the causes of its scaling with body mass are not yet fully explained. Nevertheless, observations of negative correlations between red blood cell (RBC) size and the rate of metabolism suggest that size variation of these cells responsible for oxygen supply may play a crucial role in determining metabolic rate scaling in vertebrates. Based on a prediction derived from the Cell Metabolism Hypothesis, metabolic rate should increase linearly with body mass in species with RBC size invariance, and slower than linearly when RBC size increases with body mass. We found support for that prediction in five species of eyelid geckos (family Eublepharidae) with different patterns of RBC size variation during ontogenetic growth. During ontogeny, metabolic rate increases nearly linearly with body mass in those species of eyelid geckos where there is no correlation between RBC size and body mass, whereas non-linearity of metabolic rate scaling is evident in those species with ontogenetic increase of RBC size. Our findings provide evidence that ontogenetic variability in RBC size, possibly correlating with sizes of other cell types, could have important physiological consequences and can contribute to qualitatively different shape of the intraspecific relationship between metabolic rate and body mass.

## Introduction

Despite decades of research, our understanding as to the determinants of metabolic rate scaling with body size remains unsatisfactory. This has recently become a subject of renewed intense debate among the proponents of several models put forward to explain patterns of metabolic allometry [1]–[5]. Among those most debated is the “Metabolic Theory of Ecology” predicting metabolic scaling with the power of ¾ among eukaryotes which should emerge from the geometry of resource distribution networks [1], [6]–[8]. The “Metabolic-level Boundaries” hypothesis views the interspecific variation of metabolic scaling as a consequence of species-typical lifestyles and various ecological factors that affect the overall metabolic level (intensity) of a species, and thereby the relative effects of surface area- versus volume-related processes on the scaling slope [4], [9], [10]. Kooijman [11], in his “Dynamic Energy Budget” theory abstracts the organism into two compositionally homogenous parts called “structure” and “reserve” and explains the negative allometry of metabolic rate as the result of an increase of the proportion of a metabolically inactive “reserve” with an increase of structural body mass [12]. The last of the currently most explored hypotheses is the “Cell Metabolism Hypothesis” [2], [13], which postulates that variation in metabolic scaling could be largely attributed to differences in cell size. This hypothesis predicts that larger cells are metabolically more frugal, i.e., they have lower metabolic rate per volume unit, due to their relatively smaller membrane surface to cell volume, which should determine the cell metabolic rate. The Cell Metabolism Hypothesis was supported by studies of metabolic scaling within mammalian and bird lineages [2], within a reptilian family [14], and between polyploid and diploid forms of fish from a *Cobitis* species complex [15]. In the latter two experimental studies, red blood cell (RBC) size was taken as a proxy of general cell size of an organism. However, RBC size correlates with size of cells in other tissues only in some (amphibians, passerine birds), but not the other (mammals) vertebrate lineages [16]. The negative relationship between genome size and mass-corrected metabolic rate in both passerine birds and mammals [17], [18] together with the fact that genome size strongly correlates with RBC size in both groups [19], [20] suggest that just RBC size variation may be crucially connected with metabolic scaling, possibly due to their direct role in oxygen supply. It is possible that the link between metabolic rate and cell size expected under the Cell Metabolism Hypothesis as formulated by Kozłowski et al. [2] holds in vertebrates only for RBCs rather than for organismal cells in general.

Surprisingly, very little of the current discussion on mechanisms of metabolic scaling involves the consequences of ontogenetic changes in metabolic rates to the observed patterns of scaling (but see e.g., [4], [9], [21]–[23]). Those patterns are typically analysed in adult individuals with little regard to the life-history of growth and development which underlies distributions of adult sizes. All of the aforementioned models make clear predictions with regard to the shape of ontogenetic allometries of metabolic rates. According to the Metabolic Theory of Ecology, the ¾ power scaling should hold for both interspecific and ontogenetic allometries, because it reflects the organization of transport networks that ensures minimization of costs of transportation across bodies of various sizes [24]. On the other hand, the other three hypotheses point to variability in metabolic scaling among individual species. The Metabolic-level Boundaries hypothesis focuses on the differences in ecology among species. According to this hypothesis, species with high energy expenditure (usually active species) tend to have lower scaling exponents of the relationship between metabolic rate and body mass, which could reflect amounts of energetically expensive tissues associated with different activity levels [10]. The Dynamic Energy Budget hypothesis views variability in intraspecific (ontogenetic) metabolic scaling as a consequence of surface-area-specific variation in assimilation rate and “reserve” density. However, no simple general prediction is given [12]. The Cell Metabolism Hypothesis predicts that the metabolic rate allometry should reflect the contribution of cell size increase versus cell number increase to body mass enlargement within a clade [2]. Therefore, individuals of species characterized by cell-size invariance should exhibit an isometric (linear) relationship between metabolic rate and body mass during ontogeny. Conversely, an increase of cell size during ontogeny should result in flattening of metabolic rate scaling.

Chown et al. [25] supported the aforementioned predictions of the Cell Metabolism Hypothesis for a mutual relationship between cell size contribution to body size changes and metabolic scaling at intraspecific levels in ants, where particular species differ in the proximate mechanism of body size variation among castes. However, the Cell Metabolism Hypothesis has not yet been tested in relation to ontogenetic changes of metabolic scaling. Originally, Kozłowski et al. [2] expected isometric scaling of metabolic rate during ontogeny because they assumed stable cell size within a species. This was because they expected a close relationship between cell size and genome size, the latter being fairly stable within species. It has been shown, however, that cell sizes, including RBC sizes in vertebrates, are phenotypically plastic, and they are significantly affected, for example, by temperature or nutrition [26]–[28]. Recently, Grenat et al. [29] documented an increase in the size of RBC with age and body size among juvenile and adult frogs (*Odontophrynus americanus*), though they did not test any physiological or phenotypic effects of the cell size variation. Pis [30] showed that RBC size changes during development in a bird (grey partridge, *Perdix perdix*) during a short period after hatching and demonstrated an inverse relationship between erythrocyte size and mass-specific resting metabolic rate within the first three months after hatching.

Our aim is to test the prediction of the Cell Metabolism Hypothesis as to metabolic scaling by exploring whether there exists consistent ontogenetic variation in RBC size and how this relates to metabolic scaling among closely related species. We use five species of the family of the eyelid geckos (Eublepharidae) as a model system. Previously, we demonstrated that RBC size correlates with body size across species of the family [31]. Moreover, RBC size correlates inversely with mass-specific standard metabolic rate among species of eyelid geckos, and thus their variation possibly affects interspecific metabolic scaling [14]. However, we were not able to disentangle whether RBC size variation affects metabolic scaling causally, or just due to correlation with body size across species. Therefore, here, we expand these studies to ontogenetic variation in metabolic rate and RBC size in several species of the group in the hope to find diversity in the relationship between RBC size variation during ontogenetic growth, which would allow to test the causality. The Cell Metabolism Hypothesis predicts that metabolic rate should increase linearly with body mass in species with invariance in RBC size or lack of correlation between RBC size and body mass, while an increase of RBC size with body mass during ontogeny should result in slower than linear relationship between metabolic rate and body mass or in other words in flattening of the metabolic rate scaling.

## Materials and Methods

### Experimental animals

Eyelid geckos are a monophyletic group sharing similar morphology and lifestyle. They are all largely insectivorous and mostly ground dwelling lizards which inhabit tropical and subtropical regions of Africa, Asia and North and Central America, but they differ considerably in body size and also in RBC size ([14], [31] and references there). In this study, we combined previously published data on standard metabolic rate (SMR) and RBC size in adult individuals [14] with measurements of resting metabolic rate and RBC size in fresh hatchlings and juveniles. Typically, SMR is estimated as oxygen consumption of an adult, non-reproductive, post-absorptive ectothermic animal at rest, at a temperature not inducing thermal stress. SMR reflects the metabolic costs of maintenance, whereas metabolism of juveniles also includes metabolic costs of growth (reviewed in [4]). Nevertheless, for simplicity and avoidance of multiplying terminology we refer also to our metabolic measurements on juvenile geckos as being SMR.

We chose five species: *Coleonyx elegans* Gray, 1845, *C. mitratus* (Peters, 1845), *Eublepharis macularius* Blyth, 1854, *Goniurosaurus luii* Grismer Viets & Boyle, 1999, and *G. lichtenfelderi* (Mocquard, 1897), because in these five species we were able to cover the full range of body sizes from hatchlings to fully grown adults several years of age. Animals used for the experiment were either bred in our laboratory (Charles University in Prague, Prague, Czech Republic) or they originated from the pet trade but had been acclimated to our laboratory conditions (mean temperature 26°C) for at least one year. In our breeding facilities, animals were housed individually or in small groups in glass cages or plastic boxes of appropriate size with dry or partly moist substrate (sand or coconut shell chippings), equipped with shelter and a small dish for water. Water supplemented with calcium was provided *ad libitum* and regularly enriched with vitamins E, A and D3 (Combinal E and Combinal A + D3; IVAX Pharmaceuticals, Opava, Czech Republic). Lizards were fed live crickets (*Gryllus assimilis*) dusted with vitamins and minerals (Roboran H, Univit, Czech Republic) twice per week (hatchlings and juveniles) or once per week (adults). A light cycle of 12L:12D was maintained.

### Ethics Statement

The experiment was held under approval and supervision of the Ethical Committee at the Faculty of Science, Charles University in Prague, permit number 29552/2006-30.

### Metabolic rate measurement

**Figure 1:**
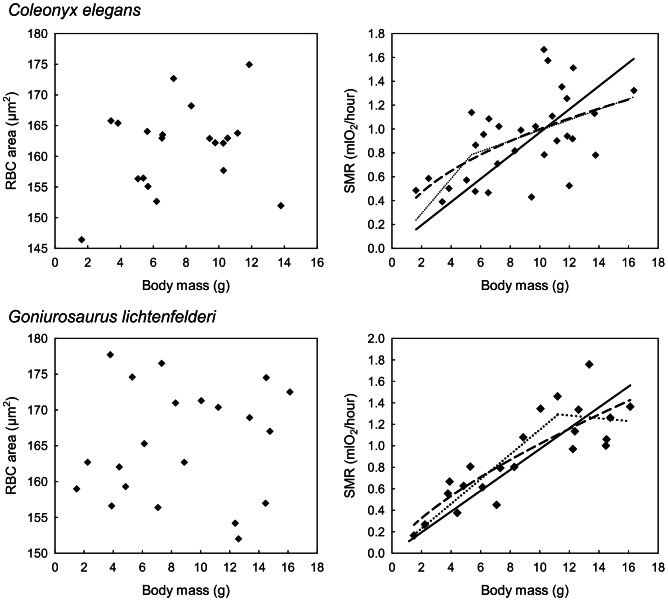
Ontogenetic changes of red blood cell size and metabolic rate with body mass. In species of eyelid geckos without correlation between red blood cell (RBC) area and body mass (left column), we cannot reject linear model as the adequate description of the ontogenetic relationships between standard metabolic rate (SMR) and body mass (right column). Each point represents a single individual. Linear function (solid line), power function (dashed line) and two-segmented linear function (dotted line) are shown. Supported models are in bold.

**Figure 2:**
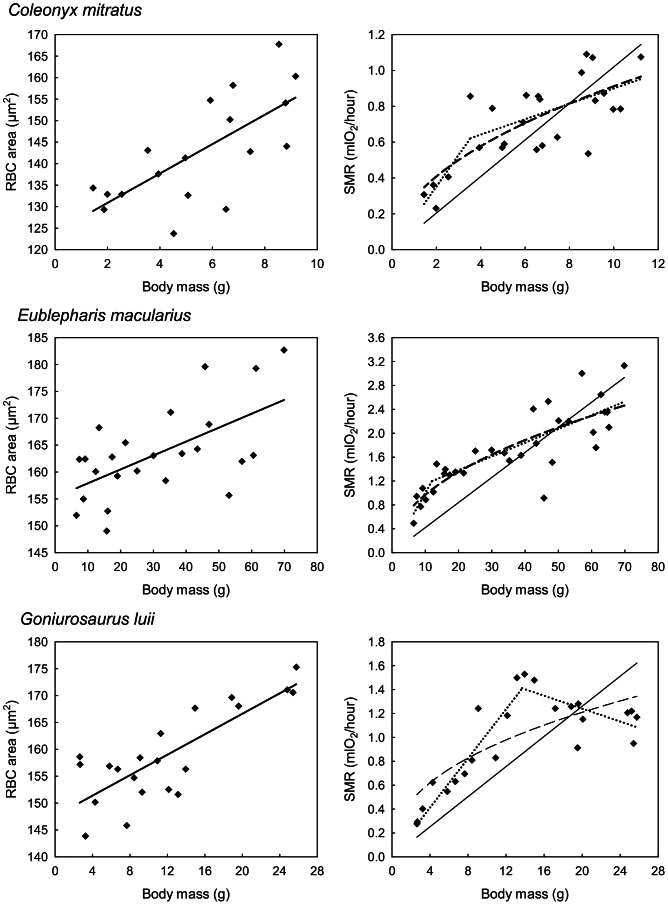
Ontogenetic changes of red blood cell size and metabolic rate with body mass. In species of eyelid geckos with correlation between red blood cell (RBC) area and body mass (left column), standard metabolic rate (SMR) increases non-linearly with body mass during ontogeny (right column). Each point represents a single individual. Linear function (solid line), power function (dashed line) and two-segmented linear function (dotted line) are shown. Supported models are in bold.

Metabolic rate was measured by the same methodology and instrumental set-up as described in Starostová et al. [14]. Briefly, SMR measurements were taken for adult or juvenile individuals at 25°C during the light phase (i.e., inactive day phase for nocturnal geckos). A stable chamber temperature was maintained by immersion of metabolic chambers into a thermally-controlled water bath (±0.1°C). Animals were fasted for two days before trials, but water was always available. Metabolic rate was measured as O_2_ consumption in a flow-through respirometry system (Sable Systems, Las Vegas, Nevada) calibrated with a bubble flow meter (Optiflow 420, Supelco, Bellefonte). To achieve high measurement precision, the animals were weighed to the nearest 0.01 g before metabolic trials and the air flow (range 5–40 ml/min) and metabolic chamber volume (50–300 cm^3^) were adjusted to the body mass of each individual. The water vapour and CO_2_ were scrubbed from the airstream prior to gas analysis. We defined SMR as the lowest 10 minutes recorded during up to 180 minutes long trials, calculated according to equation 4a of Withers [32] and converted to standard (STPD) conditions. In total, our data set consists of data on 133 measurements, with one SMR measurement per individual animal. Sample sizes and body size range for each species are presented in Tables 1 and 2 and Figs. 1 and 2.

**Table 1:** Relationship between size of red blood cells and body mass within species of eyelid geckos during ont ogeny.

Species	n	Linear regression between RBC size and BM
*Coleonyx elegans*	20	n. s., p = 0.286
*Coleonyx mitratus*	18	RBC size = 3.415*BM+124.050, p = 0.001
*Eublepharis macularius*	24	RBC size = 0.259*BM+155.320, p = 0.002
*Goniurosaurus lichtenfelderi*	21	n. s., p = 0.724
*Goniurosaurus luii*	21	RBC size = 0.952*BM+147.580, p<0.001

Number of individuals is indicated by n, red blood cells as RBC and body mass as BM.

**Table 2:** Comparison of models fitted to standard metabolic rate and body mass relationship within species of eyelid geckos during ontogeny.

Species	n	Fitted model	SMR–body mass relationship	AICc	Δ AICc	Akaike weights
*Coleonyx elegans*	31	**Linear regression**	SMR = 0.097*BM	−27.589	1.747	0.249
		**Power function**	SMR = 0.337*BM^0.472^	−29.336	0.000	0.598
		Segmented regression	SMR = 0.146*BM for BM<5.382	−26.610	2.726	0.153
			SMR = 0.044*BM+0.549 for BM≥5.382			
*Coleonyx mitratus*	25	Linear regression	SMR = 0.102*BM	−32.818	3.519	0.104
		**Power function**	SMR = 0.289*BM^0.499^	−36.337	0.000	0.602
		**Segmented regression**	SMR = 0.176*BM for BM<3.529	−34.911	1.426	0.295
			SMR = 0.043*BM+0.470 for BM≥3.529			
*Eublepharis macularius*	32	Linear regression	SMR = 0.042*BM	−15.677	8.477	0.010
		**Power function**	SMR = 0.321*BM^0.480^	−24.154	0.000	0.696
		**Segmented regression**	SMR = 0.100*BM for BM<12.043	−22.434	1.720	0.294
			SMR = 0.023*BM+0.922 for BM≥12.043			
*Goniurosaurus lichtenfelderi*	22	**Linear regression**	SMR = 0.097*BM	−25.175	0.000	0.417
		**Power function**	SMR = 0.200*BM^0.708^	−24.873	0.302	0.359
		**Segmented regression**	SMR = 0.115*BM for BM<11.201	−23.936	1.239	0.224
			SMR = −0.013*BM+1.439 for BM≥11.201			
*Goniurosaurus luii*	23	Linear regression	SMR = 0.063*BM	−17.394	14.252	0.001
		Power function	SMR = 0.349*BM^0.415^	−23.746	7.900	0.019
		**Segmented regression**	SMR = 0.103*BM for BM<13.661	−31.646	0.000	0.980
			SMR = −0.027*BM+1.781 for BM≥13.661			

Number of individuals is indicated by n, standard metabolic rate as SMR and body mass as BM. Substantially supported models with differential AICc (Δ) less than 2 and high probabilities based on Akaike weights are in bold.

### Erythrocyte size measurement

In measuring RBC size, too, we followed the methodology described in the previously published papers [14], [31], [33]. A small drop of blood was taken from the humeral vessel after completing all metabolic measurements and the blood was used to prepare blood smears. Dry smears were fixed for five minutes in methanol and subsequently dyed using standard May-Grünwald and Giemsa-Romanovski solutions. Cell size calculated as mean area of 50 dry erythrocytes per individual (104 individuals in total) was measured using light microscopy and subsequent image analysis (analySIS 1.10, Soft Imaging System).

### Data analyses

Our statistical analyses did not take into account potential sexual differences in RBC size and metabolic rate, because we were not able to determine the sex of juvenile individuals. No sexual differences were found, however, in either SMR or in RBC size for eyelid geckos in our previous studies [14], [31], [33], nor are these reported in most publications concerning reptiles (e.g., [34] and references there on SMR; [35] on RBC size).

Statistical analyses were performed using Statistica 6.0 (StatSoft, Tulsa, OK,

USA, 2001) software and the SAS 9.1.3 statistical package (SAS Institute, Cary, NC, USA, 1996). For each species, we tested the scaling of RBC size with body mass using linear regression. Ontogenetic patterns of changes in SMR with body mass were analysed by means of the NLIN procedure of SAS. The Cell Metabolism Hypothesis predicts a linear relationship between SMR and body mass across individuals without correlation between RBC size and body mass, but departure from linearity across individuals with such correlation. To test this we fitted a linear, two-segmented linear and power model to each of the data sets, where the latter two are used as a mathematical description of flattened (concave) function, which should result from nonlinearity in untransformed data. For the two-segmented linear models, we used a simple SAS program that finds the optimum breakpoint while minimizing the residual sum of squares. The power model took the form of y  =  a*(body mass)^b^, where a and b were fitted constants. Note that the power model was applied exclusively for testing non-linearity and the exponents should not be interpreted as standard scaling exponents calculated under log-log transformation. We did not log-transformed the data before all statistical analyses, because our aim was to test linearity versus non-linearity (flattening) of the SMR-body mass relationship, and transformation of data with quite narrow mass range would distort the distribution of data points. As we argued in our earlier paper [14], it is reasonable to expect that an organism with zero mass should have zero metabolism. For this reason we have run all statistical models of SMR with a zero intercept.

We then compared models' goodness of fit, expressed as the second order Akaike information criterion (AICc) score, while assuming that differences in AICc (Δ) calculated as the value of AICc for each model minus that of the model with the lowest value which are lower than 2 are not informative [36]. We calculated and compared also Akaike weights for each model.

## Results

The results of the statistical analyses are summarized in Tables 1 and 2, while scatter plots of RBC area and SMR versus body masses in the studied species are presented in Figs 1 and 2.

As indicated by the absence of statistical significance for the slopes of the relationships between RBC size and body mass, RBC size does not increase during ontogenetic growth of *C. elegans* and *G. lichtenfelderi* (Fig. 1, Table 1). In the three other eyelid gecko species (*C. mitratus, E. macularius*, and *G. luii*), by contrast, RBC size significantly increases with body size during ontogenetic growth (Fig. 2, Table 1). In those three species, RBC size increases in the course of ontogeny by 23%, 17% and 18%, respectively.

In accordance with the predictions derived from the Cell Metabolism Hypothesis, in two species characterized by the lack of ontogenetic RBC size increase – *C. elegans* and *G. lichtenfelderi* – we were not able to reject a linear model as an adequate description of the SMR scaling in favor of the models expecting nonlinearity of this relationship. The predictions of the Cell Metabolism Hypothesis are further supported by the analyses of ontogenetic SMR changes in all three species whose RBC size significantly increased in the course of ontogeny (*C. mitratus, E. macularius*, and *G. luii*). In this case relationships between SMR and body mass were significantly better fitted by two-segmented linear or power models than by simple linear models (Table 2). Mean Akaike weights for a linear model fitted to the SMR-body size data for two species with no relationship between RBC size and body size were considerably larger (0.333) than mean Akaike weights (0.038) of the linear model fitted in three species whose RBC size increases with body size during ontogeny. This points to substantial nonlinearity of the SMR-body mass relationships in the latter three species.

## Discussion

Due to the correlation between RBC size and genome size, RBC size in vertebrates used to be considered a stable species-specific characteristic [14], [31], [37], [38]. Nevertheless, RBC size is considerably plastic with respect to thermal environment [26] and ontogeny ([29], [30], this study). We demonstrated that RBC size increases with body mass through ontogeny in three species, but not in another two species of eyelid geckos. Previously, measuring RBC size in adult individuals using the same methodology, we demonstrated that RBC size increases with body mass across species of the family [31]. The increase in mean RBC size (dry projection area) from the smallest (*Coleonyx brevis*; mean adult body mass 4.6 g) to the largest (*Eublepharis angramainyu*; mean adult body mass 89 g) species came to about 36%. The ontogenetic increase of RBC size in all three species with significant correlation between RBC size and body mass during ontogeny was about 20%, although the species largely differ in absolute ranges of body mass (Fig. 2).

The variability in RBC size could have important physiological consequences. Erythrocytes serve to distribute oxygen to all other tissues, which is potentially a constraining process for oxidative metabolism. Their diameters, limited by the nuclei size and shape, necessarily determine blood capillary diameter and hence affects all tissues [39] and thus the total metabolic rate of an organism. The variation in cell size is a key component of the Cell Metabolism Hypothesis on metabolic rate scaling [2]. For ontogenetic scaling, this hypothesis expecting causal relationship between RBC size and metabolic rate, predicts a linear relationship between logarithmically untransformed SMR and body mass across individuals without correlation between RBC size and body mass, but slower than linear SMR increase relationship across individuals with such correlation (i.e., flattening of metabolic rate scaling). The pattern of ontogenetic scaling among the species of eyelid geckos described here fits the prediction well. Clear non-linearity in SMR scaling was observed only in species with significant increase of RBC size with body mass. The pattern is consistent with the single-species study in the grey partridge demonstrating negative relationship between RBC size and mass-specific metabolic rate [30].

It is notable that in the congeneric species in our study we observed the contrasting patterns in scaling of RBC size and SMR (*C. mitratus* versus *C. elegans* and *G. lichtenfelderi* versus *G. luii*), which suggests that the increase of RBC size during ontogenetic growth is not phylogenetically conservative. Analogously, two independently established clines in body size in *Drosophila subobscura* across latitudinal gradient differed in presence of correlation of cell size with body size [40]. It was suggested that the differences in cell size increase with body size among closely related species or populations of *Drosophila* could reflect whether larger body size is achieved via prolonged growth or higher growth rates [28]. The growth data in eyelid geckos suggest that differences in RBC size and metabolic rate scaling might not be correlated with differences in growth rates: three species (*E. macularius*, *C. elegans* and *C. mitratus*) with comparable growth rates [41] show different patterns in both RBC size and SMR scaling.

Metabolic rate of an animal can be affected by many factors. We were largely able to control external factors (e.g., environmental temperature) in the laboratory, but there remain many internal factors potentially responsible for shaping ontogenetic changes in SMR. The traits known to affect metabolic rate and at the same time differing between juveniles and adults include level of tissue hydration, which generally decreases with age and increases SMR [42]; relative proportions of internal organs [43], [44], many of which scale allometrically with body size [45]; density of mitochondria in muscle fibres [46], [47]; and size-correlated differences in enzyme activity [48]. Nevertheless, we demonstrate here that RBC variation contributes to differences in SMR dynamics across ontogeny. There are several possible, not mutually exclusive, mechanisms generating this pattern. First, RBC size may correlate with cell sizes in other tissues and serve as a proxy of generalized cell size as found in passerine birds [16], which shapes SMR scaling as expected by earlier versions of the Cell Metabolism Hypothesis [2]. The correlation between RBC size and size of cells in other tissues has not yet been tested in geckos. Second, RBC diameter may determine the architecture of circulatory system, namely capillary diameter. Third, RBC surface-to-volume ratio, lower in small RBC, may directly affect gas exchange [39], [49]. The role of these mechanisms requires further studies.

There is an ongoing debate whether the scaling of metabolic rate is primarily driven by constraints of supply network [1] or intrinsic demands [50]. Under the latter possibility, ontogenetic scaling of metabolic rate should be largely influenced by metabolic demands of growth and development resulting in differences of scaling among particular ontogenetic stages [22], [51], [52], [53]. We measured total metabolic rate of non-active animals without attempting to estimate the importance of particular components of energy budget. Under the Cell Metabolic Hypothesis, however, total metabolic rate, not just maintenance costs, should depend on the size variation of the cells. Therefore, the model should hold regardless of the varying contribution of costs of growth to the total metabolic rate across ontogeny.

In summary, our study further highlights that intraspecific allometries from juveniles to adults provide an important perspective on processes shaping metabolic allometries. Especially organisms exhibiting indeterminate growth such as reptiles, where there is usually substantial variation in body size between juveniles and adults (see also [54]) but where many aspects of general biology (e.g., diet) or physiology are still shared, could be informative in this respect. Here, we found support for the causal role of RBC size variation in shaping metabolic rate scaling, which is in agreement with general predictions of the Cell Metabolism Hypothesis in a reptilian lineage. During ontogeny, metabolic rate increases nearly linearly with body mass in those species of eyelid geckos where there is no correlation between RBC size and body mass (Type II pattern defined by [4]), whereas clear non-linearity of metabolic rate scaling is evident in those species with ontogenetic increase of RBC (Type III pattern [4]). The variability in cell size could have important physiological consequences, such as a qualitatively different pattern of SMR-body mass relationship.
